# Bulked segregant RNA-seq reveals complex resistance expression profile to powdery mildew in wild emmer wheat W762

**DOI:** 10.3389/fpls.2024.1387427

**Published:** 2024-05-16

**Authors:** Zejun Qian, Ruishan Liu, Xueqing Liu, Yanmin Qie, Jiangchun Wang, Yan Yin, Qingguo Xin, Ningning Yu, Jiadong Zhang, Yaoxue Li, Jiatong Li, Yintao Dai, Cheng Liu, Yuli Jin, Pengtao Ma

**Affiliations:** ^1^ Yantai Key Laboratory of Characteristic Agricultural Bioresource Conservation & Germplasm Innovative Utilization, College of Life Sciences, Yantai University, Yantai, China; ^2^ Institute of Grain and Oil Crops, Yantai Academy of Agricultural Sciences, Yantai, China; ^3^ Institute of Cereal and Oil Crops, Hebei Academy of Agricultural and Forestry Sciences/Hebei Laboratory of Crop Genetic and Breeding, Shijiazhuang, China; ^4^ Crop Research Institute, Shandong Academy of Agricultural Sciences, Jinan, China

**Keywords:** durum wheat, powdery mildew, BSR-seq, expression profiling, DEG

## Abstract

Powdery mildew, caused by *Blumeria graminis* f. sp. *tritici* (*Bgt*), is one of the most destructive fungal diseases threatening global wheat production. Exploring powdery mildew resistance (*Pm*) gene(s) and dissecting the molecular mechanism of the host resistance are critical to effectively and reasonably control this disease. Durum wheat (*Triticum turgidum* L. var. *durum*Desf.) is an important gene donor for wheat improvement against powdery mildew. In this study, a resistant durum wheat accession W762 was used to investigate its potential resistance component(s) and profile its expression pattern in responding to *Bgt* invasion using bulked segregant RNA-Seq (BSR-Seq) and further qRT-PCR verification. Genetic analysis showed that the powdery mildew resistance in W762 did not meet monogenic inheritance and complex genetic model might exist within the population of W762 × Langdon (susceptible durum wheat). After BSR-Seq, 6,196 consistently different single nucleotide polymorphisms (SNPs) were called between resistant and susceptible parents and bulks, and among them, 763 SNPs were assigned to the chromosome arm 7B. Subsequently, 3,653 differentially expressed genes (DEGs) between resistant and susceptible parents and bulks were annotated and analyzed by Gene Ontology (GO), Cluster of Orthologous Groups (COG), and Kyoto Encyclopedia of Genes and Genomes (KEGG) pathway enrichment. The potential regulated genes were selected and analyzed their temporal expression patterns following *Bgt* inoculation. As a result, nine disease-related genes showed distinctive expression profile after *Bgt* invasion and might serve as potential targets to regulate the resistance against powdery mildew in W762. Our study could lay a foundation for analysis of the molecular mechanism and also provide potential targets for the improvement of durable resistance against powdery mildew.

## Introduction

Wheat (*Triticum aestivum* L., 2*n* = 6X = 42, AABBDD) is a globally significant staple crop closely associated with food security worldwide (Li et al., 2019; Han et al., 2020). As a primary source of nutrition, wheat provides essential macronutrients such as carbohydrates, proteins, and a variety of vitamins. However, wheat production often faces the threat from various fungal diseases. Powdery mildew, caused by *Blumeria graminis* f. sp. *tritici* (*Bgt*), is one of the most devastating diseases of wheat and can significantly reduce wheat yield by 10%–15% and even up to 62% in severe cases (Singh et al., 2016; Li et al., 2019; Wang et al., 2021). Therefore, it is important and urgent to explore and utilize more novel and broad-spectrum powdery mildew resistance (*Pm*) genes to effectively control this disease.

To date, more than 100 *Pm* genes/alleles have been identified in common wheat and its relatives, including 69 officially designated *Pm* genes at 64 loci (*Pm1*-*Pm69*, *Pm8* = *Pm17*, *Pm18* = *Pm1c*, *Pm22 = Pm1e*, *Pm23 = Pm4c*, *Pm31 = Pm21*) as well as dozens of provisionally named genes (McIntosh et al., 2020; He et al., 2021; Wang et al., 2023; Li et al., 2024). Among them, the genes derived from common wheat can be directly introduced into susceptible cultivars for resistance breeding, such as *Pm1a* (Sears and Briggle, 1969), *Pm3b* (Briggle, 1969), *Pm5e* (Huang et al., 2003), *Pm24* (Xue et al., 2012), *Pm38/Lr34/Yr18/Sr57* (Krattinger et al., 2009), and *Pm46/Yr46/Lr67/Sr55* (Moore et al., 2015), but these genes often have low genetic diversity compared to wheat cultivars and are easy to be defeated after long-term of promotion in production. In comparison, the genes originated from the wheat relatives often possess higher genetic variations and exhibit strong ability to withstand *Bgt* variations, such as *Pm12* from *Aegilops speltoides* Tausch (Zhu et al., 2023) and *Pm21* from *Dasypyrum villosum* L. Candagy (He et al., 2018) and, hence, have higher value in wheat improvement against powdery mildew in the future (Han et al., 2024a, Han et al., 2024b, Han et al., 2024c). Meanwhile, more and more *Pm* genes have gradually lost their resistance to powdery mildew and also many effective *Pm* genes are difficult to be utilized in breeding due to linkage drags and other negative effects in the breeding practices. For example, *Pm1a* and *Pm8* have been widely used in wheat production and breeding for many years, but their resistance have gradually lost in all or part of wheat planting areas due to the continuous variation of powdery mildew pathogens (Cowger et al., 2018). The *Pm12* was not only highly resistant to powdery mildew but also accompanied by poor yield and quality traits, making it difficult to directly utilize in breeding (Zhu et al., 2023). Therefore, mining more novel effective *Pm* genes and introducing them into wheat cultivars are significant for wheat production and disease resistance breeding.

Durum wheat (*T. turgidum* L. var. *durum* Desf., simply *T. durum*, 2*n* = 4*X* = 28, AABB) is a tetraploid wheat species and often possesses multiple resistances to leaf rust, stem rust, stripe rust and powdery mildew (Miedaner et al., 2019). Four *Pm* genes have been identified in the past decades, including *Mld*, *Pm3h*, *PmDR147*, and *Pm68*. Among them, *Mld* is a recessive gene located on chromosome 4B which could be solely used or pyramided with other *Pm* genes in wheat breeding (Bennett, 1984); *Pm3h* is a dominant resistance gene located on chromosome arm 1AS, probably originates from an Ethiopian durum wheat accession (Srichumpa et al., 2005); *Pm3h* was also originally identified in durum wheat, and subsequently confirmed to be same as *Pm3d* after cloning (Yahiaoui et al., 2006); *PmDR147* is also a dominant gene mapped on chromosome arm 2AL in durum wheat accession DR147 (Zhu et al., 2004).

Bulked segregant RNA-Seq (BSR-Seq), which combined the bulked segregant analysis (BSA) and RNA sequencing (RNA-Seq), is a high-efficiency strategy in genomics research of the complex polyploid species. In this strategy, RNA-seq is independent on pre-existing databases of expressed genes and can provide an unbiased view of gene expression profiling (Pearce et al., 2015; Pankievicz et al., 2016; Han et al., 2024a), thus is an effective and low-cost method to comprehensively evaluate the gene expression pattern of the *Pm* genes after inoculation by *Bgt* isolates. Additionally, BSR-seq can also overcome the adverse effects of the genome sequences and obtain sequence and expression information of almost all transcripts of a specific cell or tissue in a certain stage, so it is an efficient method for rapid gene mapping (Wang et al., 2017; Hao et al., 2019), especially for the crop species with complex genomes, such as wheat and its relatives (Zhang et al., 2017).

W762 is a durum wheat accession that shows high resistant to powdery mildew at the whole stage. To dissect its genetic basis against powdery mildew, in this study, we intended to (i) clarify the genetic pattern of powdery mildew resistance, (ii) identify differentially expressed genes (DEGs) at the whole-genome scale, and (iii) profile the expression of the key genes associated with resistance to powdery mildew. Our study could lay a foundation for analysis of the molecular mechanism and also provide potential targets for the improvement of durable resistance against powdery mildew.

## Materials and methods

### Plant materials and pathogens

The durum wheat accession W762, provided by International Maize and Wheat Improvement Center (CIMMYT), was used to test its reaction pattern against powdery mildew. The susceptible durum wheat accession Langdon (LDN), also provided by CIMMYT, was crossed with W762 to produce F_1_, F_2_, and F_2:3_ generations for genetic analysis and BSR-Seq analysis. Wheat cultivar Mingxian 169, which was susceptible to all the *Bgt* isolates tested in this study (Ma et al., 2018), was used as the susceptible control in phenotypic assessment experiment. Thirty-two *Bgt* isolates B05, E07, E09, E15, E17, E18, E20, E21, E23–1, E31, F01, F02, F03, F05, F06, F07, F08, F09, F10, F11, F13, F16, F17, F18, F19, F21, F22, F23, F24, F25, F28, and F32, provided by Prof. Hongxing Xu, Henan University, Kaifeng, China and Prof. Yilin Zhou, Institute of Plant Protection, Chinese Academy of Agricultural Sciences, Beijing, China, were used to evaluate the resistant spectrum of W762. These *Bgt* isolates were previously and preserved on the susceptible seedlings which were put in independent glass tubes with three layers of gauze to avoid cross infection.

### Phenotypic assessment to different *Bgt* isolates

Phenotypic assessment of W762 to the 32 *Bgt* isolates was determined in the greenhouse of Yantai University, Yantai, China. At least five seeds of W762 and LDN were sown in a 128-cell (3.2 cm× 3.2 cm× 4.2 cm) rectangular tray (54 cm× 28 cm× 4.2 cm) and the susceptible control Mingxian 169 was planted randomly in the trays. These trays were put in an independent growth chamber separately to be infected with different *Bgt* isolates. When the seedlings grown to the two-leaf stage, all the seedlings were inoculated with fresh conidiospores increased on Mingxian 169 seedlings and incubated in a chamber at 18°C for 24h with 100% humidity and then cultivated with a daily cycle of 14h of light at 22°C and 10h of darkness at 18°C. After 10–14 days, the spores were fully developed on the first leave of susceptible check Mingxian 169. Infection types (ITs) on each plant were assessed on a 0–4 scale, with IT 0, 0; 1, and 2 being regarded as resistant, and IT 3 and 4 as susceptible (Si et al., 1992; Wang et al., 2009). All tests were repeated three times to assure the reliability of the data.

### Microscopic analyses of reaction process after *Bgt* invasion

Microscopic analyses were performed as previously described (Wang et al., 2014). The 2 cm leaf segments were cut at 0h, 0.5h, 2h, 4h, 12h, 24h, 36h, 48h, and 72h after inoculating the *Bgt* isolate E09 and immediately fixed at 37°C for 24h in 2 ml of Carnoy’s Fluid (ethanol: acetic acid, 3:1, v/v), then stained with 2 ml of 0.6% (w/v) Coomassie blue solution for 3 min. Excess dye was rinsed off carefully with distilled water. Samples were observed under an Olympus BX-53 microscope (Olympus, Japan).

### Genetic analysis and preparation of samples for BSR-Seq

To determine the inheritance of powdery mildew resistance in W762, the *Bgt* isolate E09, a prevalent *Bgt* isolate in North China (Zhou et al., 2005), was selected to inoculate W762, LDN, and their F_1_, F_2_, and F_2:3_ progenies for genetic analysis. After phenotypic evaluation, the numbers of resistance and susceptible plants were counted, and then a goodness-of-fit assessment was performed to determine the resistant/susceptible ratio using a chi-squared (χ^2^) test. The deviations of the observed phenotypic data from the theoretically expected segregation ratios were then evaluated using the SPSS 16.0 software (SPSS Inc., Chicago, United States) at *p* < 0.05.

More than 20 seeds of each F_2:3_ family were sown for further genetic analysis and preparation of the samples for BSR-Seq. Resistant and susceptible RNA bulks were constructed by separately mixing equal amounts of RNA from the 30 homozygous resistant and susceptible F_2:3_ families, respectively. When the spores were fully developed on the first leaves of Mingxian 169, the total RNA of W762, LDN, resistant, and susceptible RNA bulks were extracted from the young leaves using TRIzol reagent (Invitrogen, Carlsbad, California, USA) following the manufacturer’s recommendations.

### BSR-Seq analysis

First, the RNA samples underwent quality and integrity testing. The eligible mRNA was isolated from total RNA by using Oligo (dT) magnetic beads paired with poly(A) tails of mRNA through A-T complementary nature. Then, the mRNA was randomly fragmented by adding the fragmentation buffer. The cDNAs were synthesized based on the mRNA template, random hexamers, dNTPs, buffer, and DNA polymerase I. After cDNA purification, end reparation, 3′ add A-tail, and sequencing adaptors ligation, fragment sizes were selected with AMPure XP beads. Finally, a cDNA library was obtained through polymerase chain reaction (PCR) amplification. After passing the library inspection, high-throughput sequencing was performed using the platform of Illumina HiSeq 4000 in Beijing Biomics Technology Co. Ltd. (Beijing, China). The sequencing indicator was set as 10 Gb clean data for the parents W762 and LDN and 20 Gb clean data for the bulks. After filtering on raw reads, and removing the adaptors and low-quality reads using software Trimmomatic v0.36 (Bolger et al., 2014) with default parameters, the clean reads were obtained. The high-quality reads were aligned to the Chinese Spring reference genome sequence v2.1 (RefSeq v2.1) (Zhu et al., 2021) and its annotation files by using software TopHat2 (Dobin et al., 2013). The mapped reads were used for further analysis. The read alignments were masked for PCR duplications and split for reads spanning introns before they were used to call SNPs and InDels using module “HaplotypeCaller” of software GATK v3.6 (McKenna et al., 2010). The resulting SNPs and InDels with sequencing depth less than four were abandoned, and the remaining ones were used for BSA analysis. Only variants with *P*-value of Fisher’s exact test on read count data < 1e^−8^ and allele frequency difference (AFD) > 0.6 were considered to be related to powdery mildew resistance. The RefSeq v2.1 was further used as a reference to call SNPs and InDels.

### Identification and statistics of DEGs

The genes expression levels were evaluated using FPKM (fragments per kilo base of transcript per million fragments mapped) (Trapnell et al., 2010). Using the software EBSeq (http://www.bioconductor.org/packages/release/bioc/html/EBSeq.html), DEGs were identified based on the standard of error detection rate (EDR) <0.01 and fold change (FC; ratio test/common reference) ≥2. The statistical significance of DEGs was performed using multiple tests and EDR was adjusted with the Benjamini–Hochberg procedure (Reiner et al., 2003).

### Functional annotation and enrichment analysis

The DEGs, which showed consistent expression difference between the resistant and susceptible parents and bulks, were annotated on the platform WheatOmics 1.0 (http://202.194.139.32/). Then, Gene Ontology (GO), Cluster of Orthologous Groups (COG), and Kyoto Encyclopedia of Genes and Genomes (KEGG) pathways enrichment analysis of DEGs were performed using an R package referred to Sherman et al. (2022), and among them, significance enrichment analysis for KEGG pathway was performed on the DEGs to further determine the signal transduction pathway(s) that these DEGs may be involved in.

### Quantitative real-time polymerase chain reaction

The seedlings of W762 and LDN were inoculated with the *Bgt* isolate E09 at the two-leaf stage. Then, the first leaves were sampled at 0h, 0.5h, 2h, 4h, 12h, 24h, 36h, 48h, and 72h post-inoculation (hpi) for RNA extraction using TRIzol reagent (Invitrogen, USA) following the manufacturer’s recommendations. The FastKing gDNA Dispelling RT SuperMix kit (Tiangen, Beijing, China) was employed to remove residual DNA and synthesize the corresponding cDNA using the following PCR procedure: 42°C for 15 min and 95°C for 3 min. The qRT-PCR procedure was performed using SYBR Premix Ex Taq (Takara, Beijing, China) on the Bio-Rad CFX Connect real-time PCR system (BIO-RAD, USA). The expression pattern of each gene was calculated as a fold change using the comparative CT method (Livak and Schmittgen, 2001). For each sample, three technical replications were set. The gene *TaActin* was used as the internal control for normalization.

## Results

### Powdery mildew resistance evaluation and its genetic analysis

After inoculated with 32 *Bgt* isolates, W762 conferred resistance to 10 isolates with IT 0, six isolates with IT 1, and five isolates with IT 2, whereas 11 isolates with ITs 3 and 4 (**Table 1**), which suggested the resistance to powdery mildew is moderate.

**Table 1:** Seedling infection types of W762 and Langdon to 32 different *Blumeria graminis* f. sp. *tritici* (*Bgt*) isolates.

*Bgt* isolates	W762	Langdon	*Bgt* isolates	W762	Langdon
B05	1	4	F08	4	4
E07	1	4	F09	0	4
E09	0	4	F10	1	4
E15	3	4	F11	4	4
E17	0	4	F13	2	4
E18	1	4	F16	2	4
E20	1	4	F17	0	4
E21	2	4	F18	4	4
E23–1	3	4	F19	4	4
E31	0	4	F21	1	4
F01	4	4	F22	4	4
F02	0	4	F23	0	4
F03	2	4	F24	0	4
F05	4	4	F25	0	4
F06	3	4	F28	2	4
F07	0	4	F32	4	4

Infection type (IT) described as Si et al. (1992), and 0–4 scale was used to score the infection types: 0, 0; among which 1 and 2 were regarded as resistant phenotypes, 3 and 4 were susceptible phenotypes.

**Figure 1:**
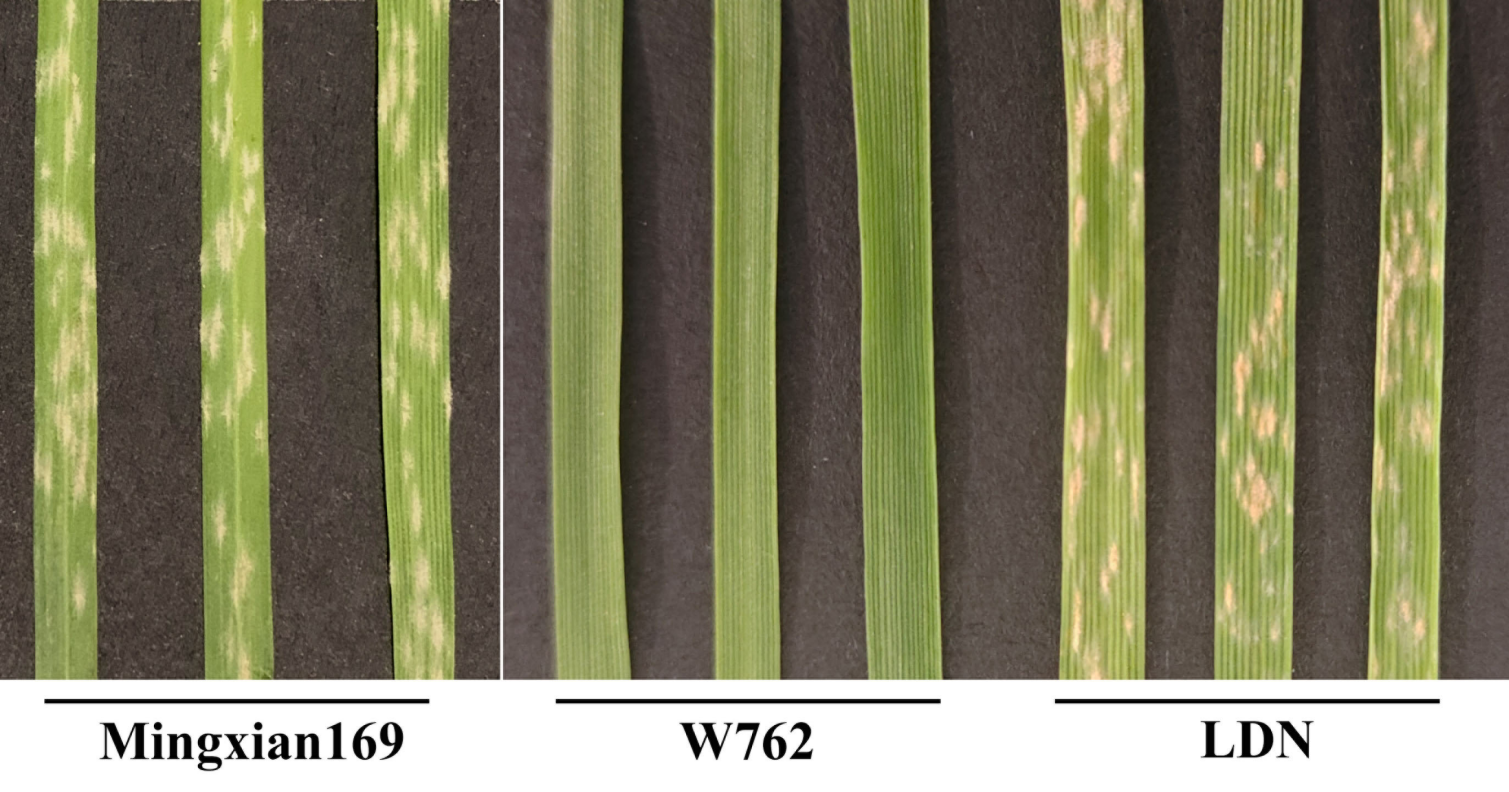
Reaction patterns of susceptible control Mingxian 169, durum wheat accessions W762, and Langdon (LDN) to the *Blumeria graminis* f. sp. *tritici* (*Bgt*) isolate E09 at the seedling stage.

**Figure 2:**
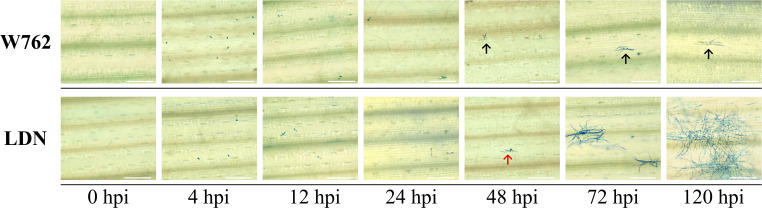
Infection process of the *Blumeria graminis* f. sp. *tritici* (*Bgt*) isolate E09 on the first leaves of W762 and Langdon (LDN). Wheat leaf samples were taken at different hpi for Coomassie blue staining. Bar = 200 μm. The black arrows indicate deformed appressorium, and the red arrows indicate normal appressorium.

Then, W762 was crossed with LDN to obtain F_1_, F_2_, and F_2:3_ progenies. When inoculated with the *Bgt* isolate E09, W762 showed no visible symptoms on the first leaves (IT 0). In contrast, the susceptible parent, LDN, had abundant sporulation which covered an area of more than 50% of the first leaves (IT 4) (**Figure 1**). Coomassie blue staining also showed large number of spores produced in LDN, and meanwhile had very mild cell death (**Figure 2**). All the F_1_ plants of W762 × LDN showed resistance at the level of IT 0 as similar as W762. Among the 200 F_2_ plants, the segregation ratio of the resistant (66) and susceptible (134) individuals did not fit for 3:1, the theoretical Mendelian segregation ratio for monogenic inheritance. The F_2:3_ families segregated 31 homozygous resistant: 94 segregating: 75 homozygous susceptible, also not fitting for the ratio 1:2:1 with monogenic inheritance. Therefore, the powdery mildew resistance in W762 did not meet monogenic inheritance and complex genetic model might exist within this population.

### Summary of the RNA-Seq data

After filtering low-quality reads and adaptors, the clean reads of 26,390,677,800 (W762), 34,391,335,500 (LDN), 40,197,188,700 (resistant bulks), and 38,205,766,800 (susceptible bulks) were obtained, respectively. The data size was more than the transcript size of the wheat genome. Therefore, it was considered to cover most expressed genes in the wheat genome. The percentage of clean reads with a Q30 was greater than 94.00% and a Q20 was greater than 97.00% for all the four samples, and the GC content ranged from 54.02% to 55.48%. After mapping the four sets of clean reads to RefSeq v2.1 individually, the percentage of reads mapping to the reference genome ranged from 81.51% to 83.31%, and the coverage of uniquely mapped reads was 99.16%–99.33%. In conclusion, the sequencing quality was high and suitable for subsequent analysis.

### Confirmation of candidate intervals

**Figure 3:**
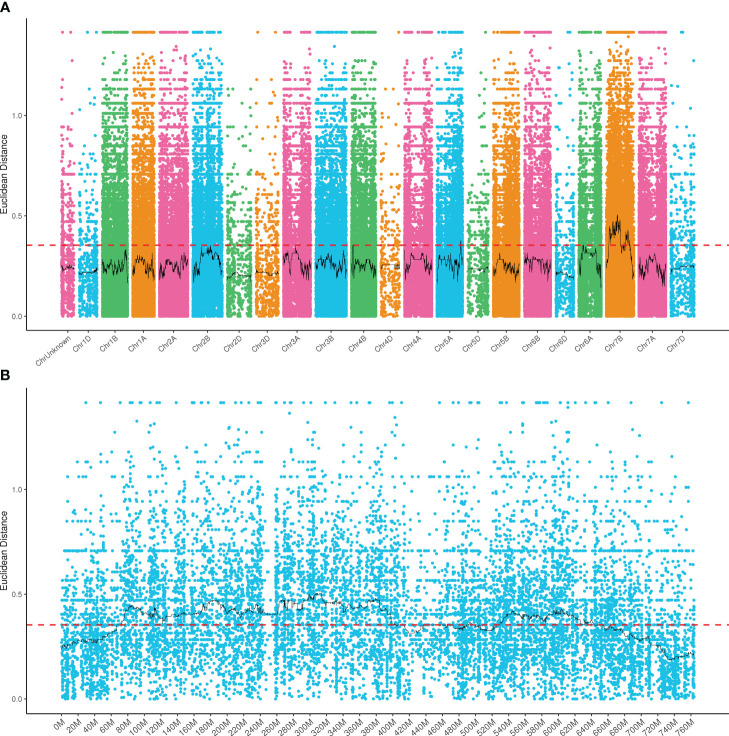
Distribution of the SNPs with consistent differences between the resistant parent W762 and susceptible parent Langdon (LDN) and their derived resistant and susceptible bulks on 21 chromosomes **(A)** and chromosome 7B **(B)**.

To evaluate the candidate intervals associated with the powdery mildew resistance, a total of 63,641, 727,819, 52,093, and 64,438 SNPs were detected from the clean data of W762, LDN, resistance and susceptible bulks, respectively. Among them, 6,196 SNPs were confirmed to be consistently different between the resistant and susceptible parents and bulks, and used for subsequent SNP index analysis. Using 99% confidence as the threshold, the putative candidate regions of 66,805,294–404,438,437 and 525,703,735–645,084,093 on chromosome 7B were identified (**Figure 3**). In this interval, 763 SNPs were identified to be consistently different between resistance and susceptible parents and bulks, account for a proportion of 34.7%. This revealed a high confidence of these candidate regions. These SNPs were used for subsequent DEGs analysis.

### Analysis of DEGs at the whole-genome scale

**Figure 4:**
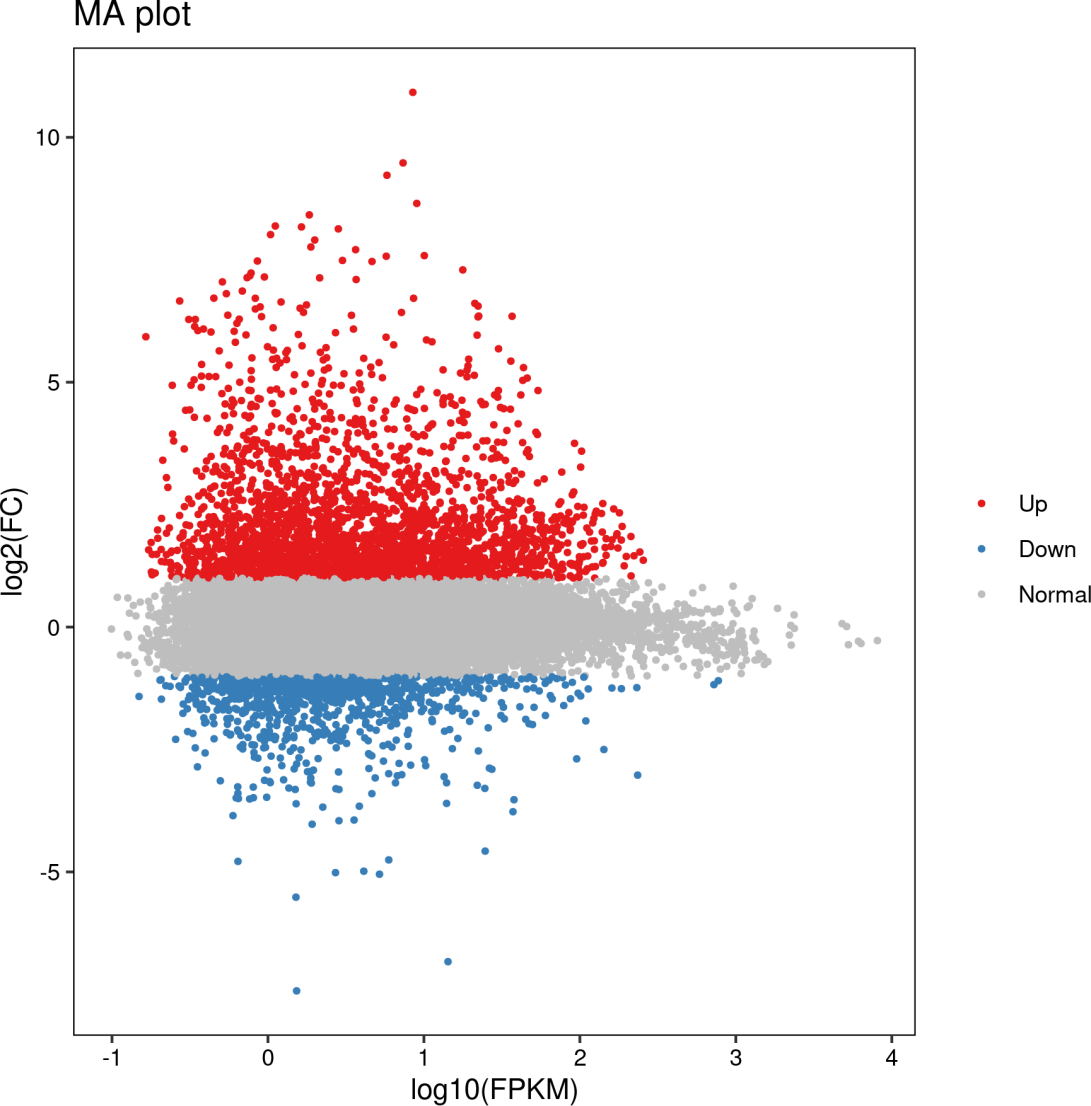
M-versus-A (MA) plot of the differentially expressed genes (DEGs) with consistent differences between the resistant parent W762 and susceptible parent Langdon (LDN) and their derived resistant and susceptible bulks. *X* and *Y* axes showed overall signal strength and output difference of the DEGs between resistant and susceptible parents and bulks.

A total of 124,200 genes were identified from the parents and bulks after BSR-Seq. Among them, 10,431 DEGs were detected between parents W762 and LDN, of which, 5,512 ones were upregulated and 4,919 ones were downregulated compared to LDN (**Figure 4**). Furthermore, 9,088 DEGs were detected between the resistance and susceptible bulks, of which 4,803 and 4,285 DEGs were downregulated and upregulated, respectively. For further screening, 3,653 DEGs showed consistent expression difference between the parents and bulks (**Figure 4**). Combined with the candidate interval analysis, only 21 DEGs were located in this interval. These genes were considered as prime candidates in the resistance response pathway to powdery mildew in W762.

**Figure 5:**
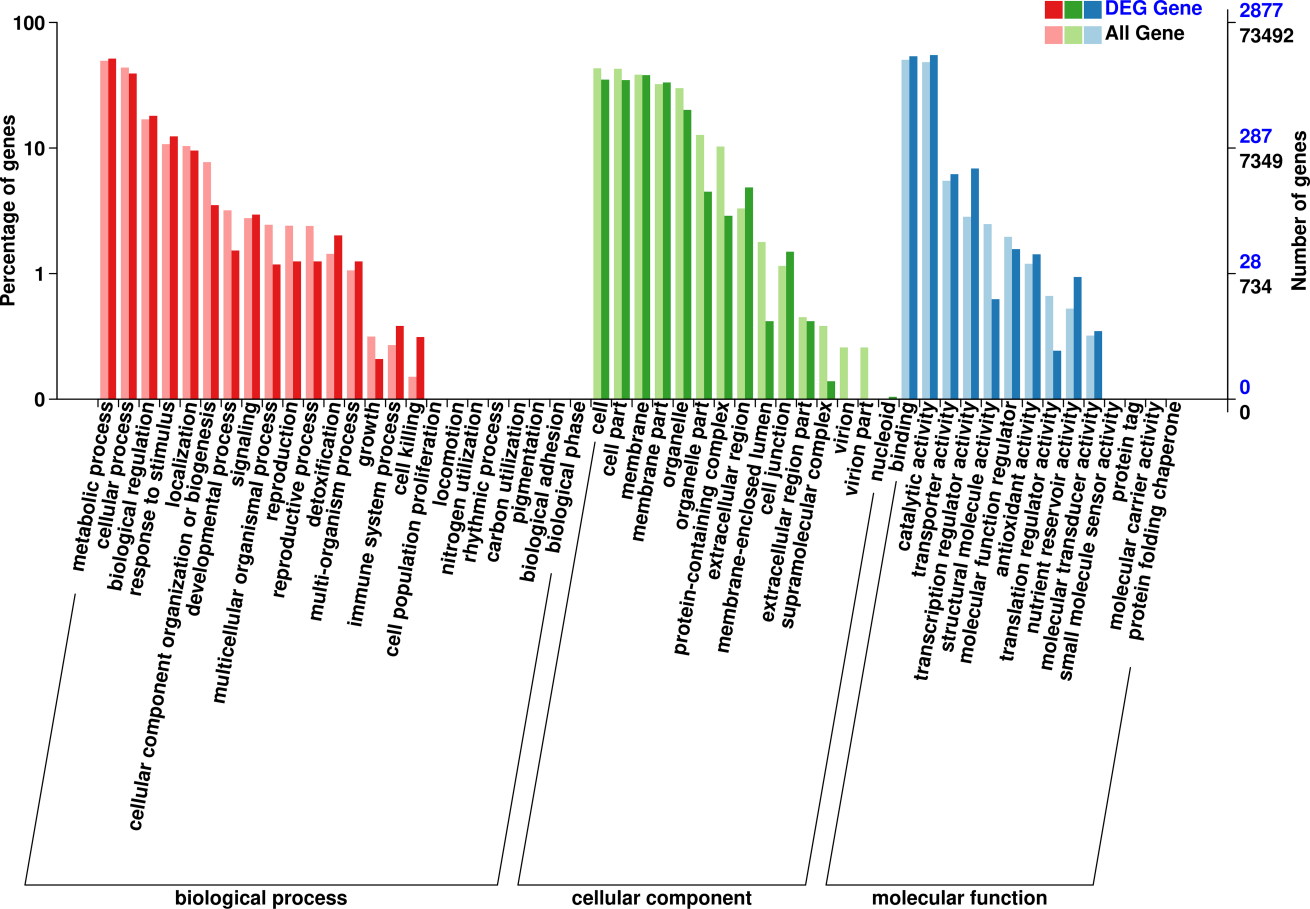
Gene ontology (GO) analysis of the differentially expressed genes (DEGs) with consistent differences between the resistant parent W762 and susceptible parent Langdon (LDN) and their derived resistant and susceptible bulks.

**Figure 6:**
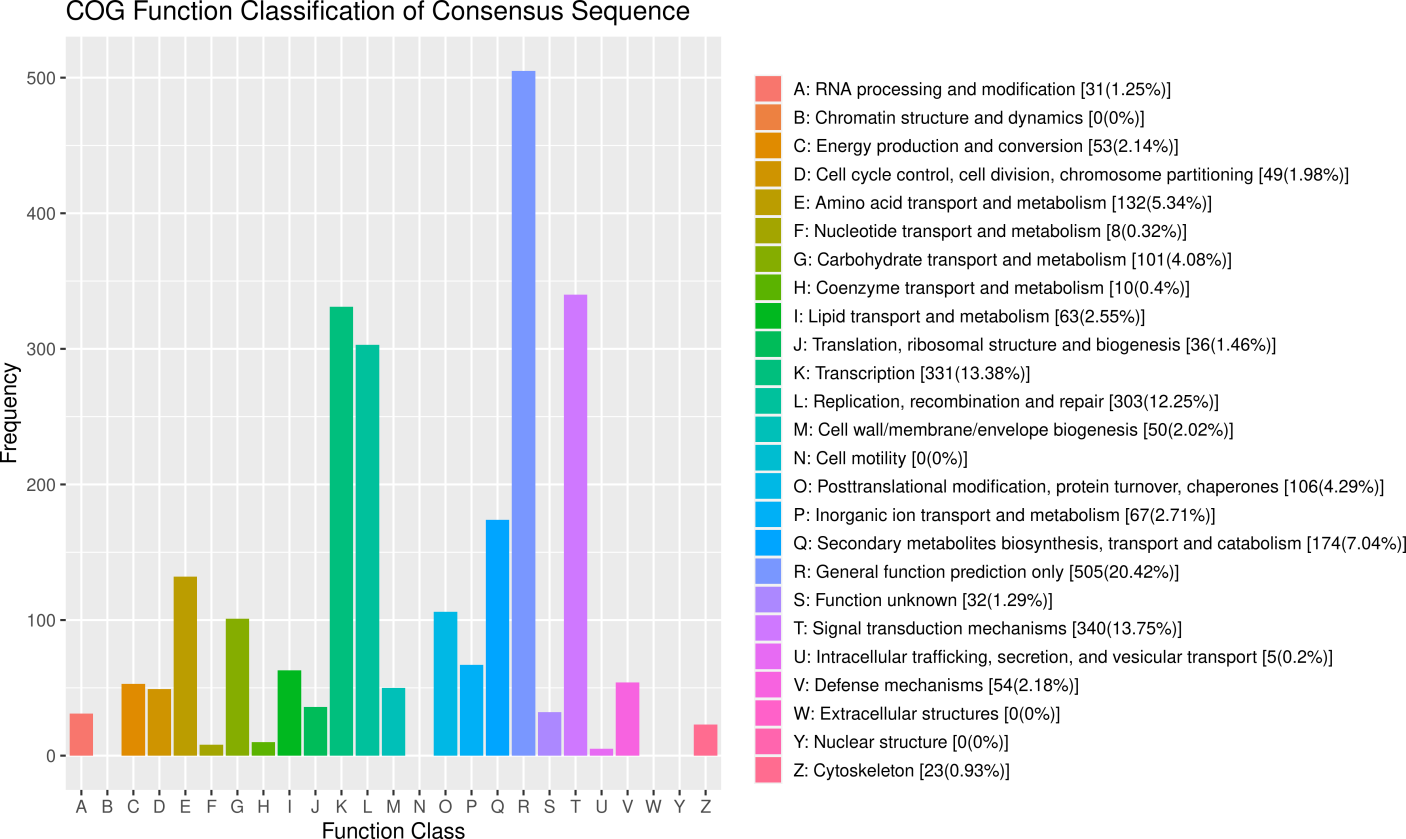
Clusters of orthologous groups (COG) analysis of the differentially expressed genes (DEGs) with consistent differences between the resistant parent W762 and susceptible parent Langdon (LDN) and their derived resistant and susceptible bulks.

After GO analysis, the DEGs were mainly involved in three branches: biological processes, cellular components and molecular function. Among them, biological processes included metabolic processes and cellular processes; cellular components included cells, cell parts, membranes, membrane’s part and organelles; and molecular functions contained binding and catalytic activity (**Figure 5**). However, the results of the GO analysis mainly focused on the processes after *Bgt* inoculation. The “response to stimuli” process was significantly enriched and may be involved in disease defense, but no known DEGs related to defense mechanism(s) were detected. Therefore, cluster of COG analysis was carried out using the same DEGs above mentioned. The data showed that the DEGs were mainly involved in signal transduction mechanisms (13.75%), transcription (13.38%), and replication, recombination and repair (12.25%). Among them, a few DEGs were directly involved in defense mechanisms, but accounting for only 2.18% (**Figure 6**). These results demonstrated that except for defense-related genes themselves, genes related to biological metabolism and synthesis also responded to the biological defense process.

**Figure 7:**
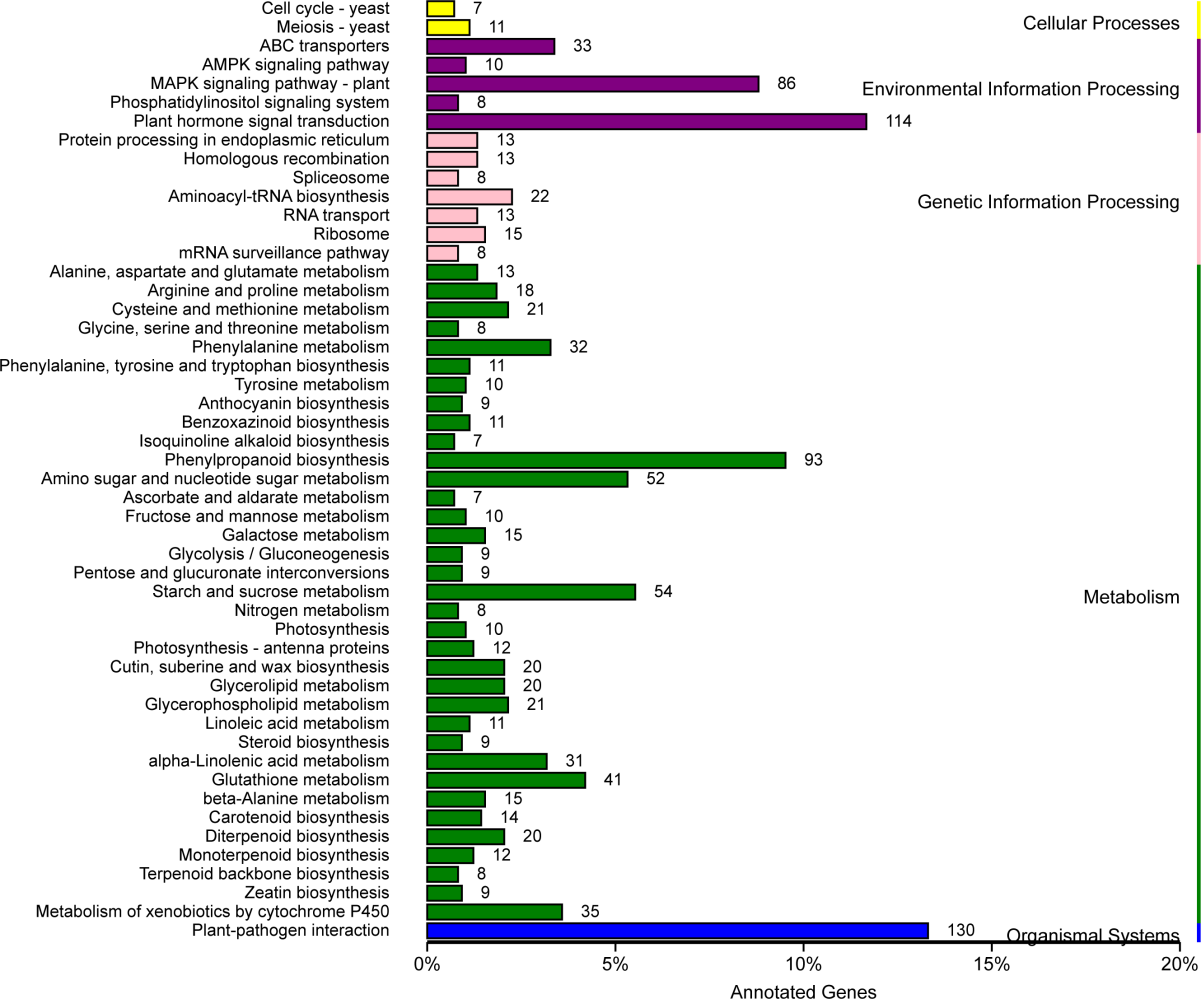
Kyoto Encyclopedia of Genes and Genomes (KEGG) pathways enrichment analysis for differentially expressed genes (DEGs) with consistent differences between the resistant parent W762 and susceptible parent Langdon (LDN) and their derived resistant and susceptible bulks.

**Figure 8:**
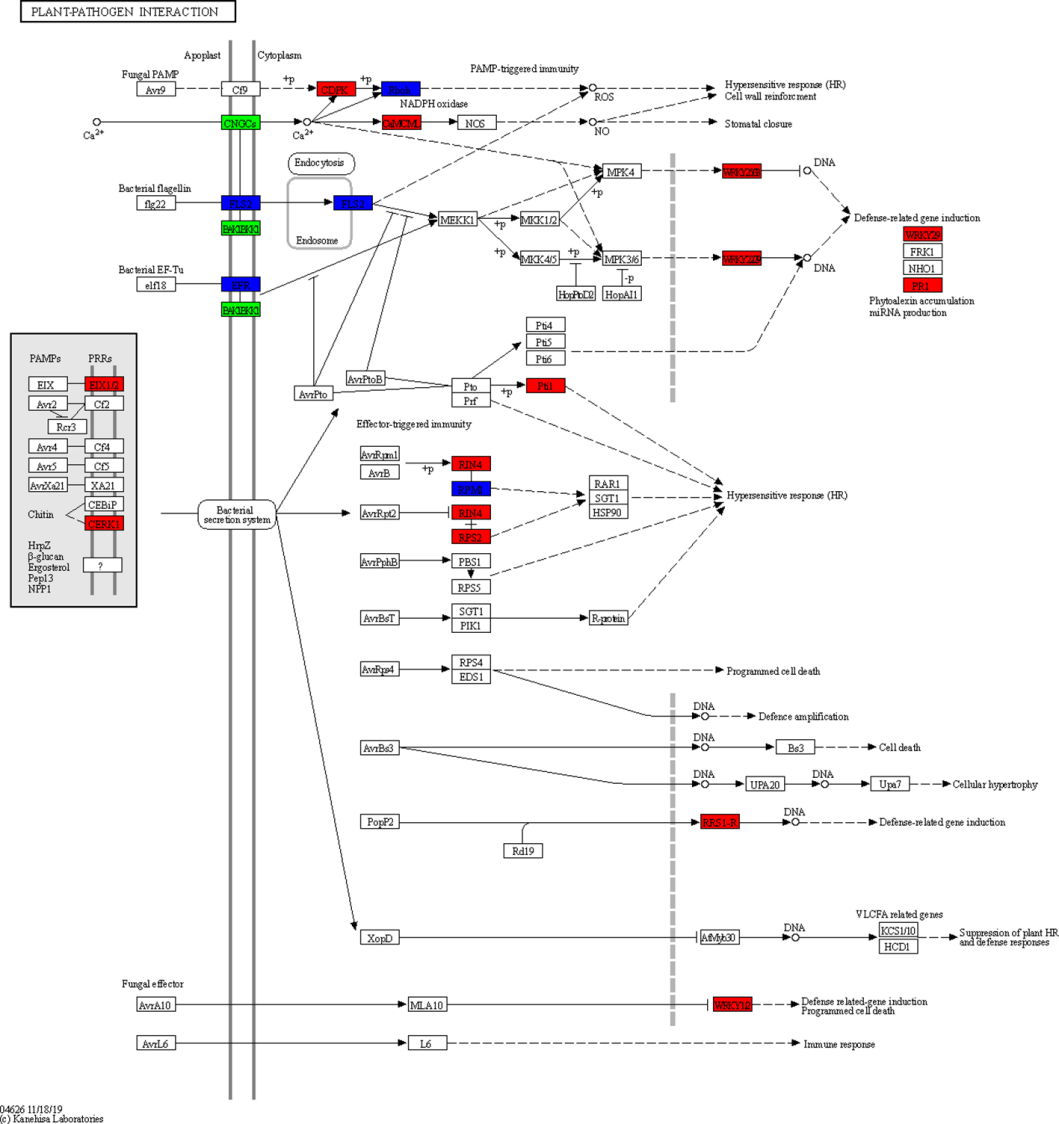
Plant-pathogen interaction pathway enriched from differentially expressed genes (DEGs) with consistent differences between the resistant parent W762 and susceptible parent Langdon and their derived resistant and susceptible bulks.

After KEGG analysis, a total of 50 pathways were significantly enriched using these DEGs, involving cellular processes, environmental adaptation processing, genetic information processing, metabolism, and organismal system. Among them, a plant-pathogen interaction pathway as well as a plant hormone signal transduction pathway emerged (**Figures 7**, **8**). These genes might be potential candidates for understanding the interaction between pathogen and plants and were also the candidate targets for molecular mechanism against wheat powdery mildew.

### Expression pattern of the key genes related to the powdery mildew resistance in W762

**Figure 9:**
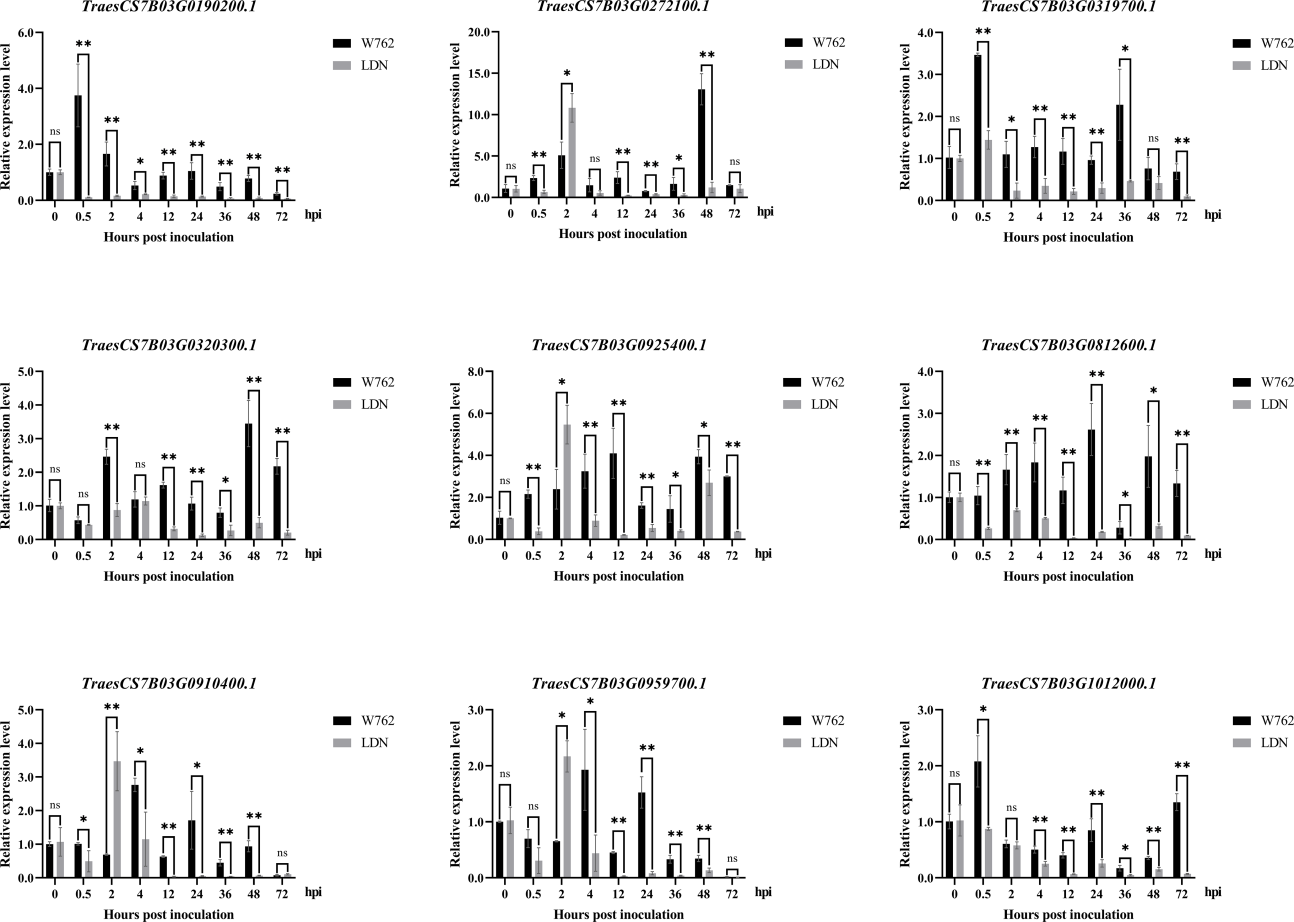
Expression profiles of nine genes in W762 and Langdon (LDN) after inoculating with the *Blumeria graminis* f. sp. *tritici (Bgt)* isolate E09 at 0, 0.5, 2, 4, 12, 24, 36, 48 and 72 hours post inoculation (hpi). Normalized values of target genes expression relative to *TaActin* were given as mean ± SD from three replicates. Statistically significant differences (Student’s t-test): *, *P* < 0.05; **, *P* < 0.01. ns: not significant.

To screen and verify the potential candidate genes responding to *Bgt* invasion, we monitored the infection process using the *Bgt* isolate E09. From the microscopic analyses of reaction process after *Bgt* invasion, we can see that 0 hpi can be used as blank control, 2–4 hpi as primary germ tube formation, 12 hpi as penetration, 24 hpi as haustorium formation, 48 hpi as secondary penetration, and 72 hpi as microcolony formation (**Figure 2**). Then, we designed the point-in-time for sampling based on the process of microcolony formation, and monitored the transcription levels of 18 potential target genes (including 12 DEGs in the candidate intervals) at different time after inoculating with the *Bgt* isolate E09. Nine genes showed significant differences expression patterns between W762 and LDN. Among them, *TraesCS7B03G0190200.1* and *TraesCS7B03G0320300* encoding serine/threonine protein kinases, *TraesCS7B03G0319700.1* and *TraesCS7B03G1012000.1* encoding protein kinase domain, *TraesCS7B03G0910400.1* encoding disease resistance protein expressed at low levels in LDN, while upregulated in W762 from 0.5 hpi, indicating that they were likely to play roles at the early stage in fighting *Bgt* invasion. *TraesCS7B03G0812600* encoding transcription factor expressed at high level at almost all the invasion process and can be considered as a key gene in the process of fighting *Bgt* invasion. While, *TraesCS7B03G0272100.1* encoding a leucine rich repeat (LRR) protein, *TraesCS7B03G0925400.1* encoding a homologous gene of *Arabidopsis thaliana RPM1* protein, *TraesCS7B03G0959700.1* encoding serine/threonine protein kinases, and *TraesCS7B03G0812600.1* encoding transcription factor expressed after *Bgt* invasion in both W762 and LDN, and reached the peak at 2 hpi in LDN (**Table 2**; **Figure 9**), suggesting that they may be involved in other pathways related to *Bgt* invasion. Therefore, these genes and their expression patterns can be used as valuable references for dissecting the genetic and molecular basis of the powdery mildew resistance in W762.

**Table 2:** Partial candidate genes of W762 on chromosome 7B and the primers for quantitative real-time PCR (qRT-PCR).

Gene	Physical genomic location	Functional annotation	Forward primer(5′–3′)	Reverse primer(5′–3′)
*TraesCS7B03G0190200*	77775536.77786594	Belongs to the protein kinase superfamily	AACGCGGTGATGGAGACTGT	CCGACGGCTGCTTCTTGGT
*TraesCS7B03G0272100*	122453467.122459516	LRR-repeat protein	GTCGTGCTGTCCTTTCTGC	ATAATGCGTAGGGTGGGTG
*TraesCS7B03G0319700*	147090873.147093400	Protein kinase domain	AAGCGTCCCGTCTTCTCCCT	TGTTCTTCTGTTTGCCACCACC
*TraesCS7B03G0320300*	147700331.147707756	Serine/Threonine protein kinases	GACTGGTGGACCTTTGGTA	GCTCACGACTGGGTATTCT
*TraesCS7B03G0345700*	163329488.163332909	disease resistance protein	ATACCTCCTCATCAAGCAA	ATCCCACAATAGATACCACTT
*TraesCS7B03G0378900*	189804519.189808558	Protein kinase domain	TATGTCCTACGGCGTCCTGG	TCGTTGGCTCTGCGTTGAT
*TraesCS7B03G0446100*	241732753.241736063	LRR-repeat protein	CACCAAGGGCGTCAAGCAG	GGGAACACCCAGGCACAGAT
*TraesCS7B03G0506300*	299127491.299151515	Protein kinase domain	GCGTCAACTTCTGCGACAT	TGCTGTTCCCAACCGAGTA
*TraesCS7B03G0532100*	327508868.327510511	Zinc finger CCCH domain-containing protein	GCCCGTATGTGACAAACTGC	CATCCCTCCTCGGTGTAGAA
*TraesCS7B03G0569800*	370388905.370393531	Serine/Threonine protein kinases	CGAGGATTGAAGTATGTGC	AAGTCAGTCTCAGTGGTGG
*TraesCS7B03G0577700*	378060043.378068505	Serine threonine-protein kinase Rio1	ACGAGCCACCAGCGGATAA	TCCTTCCGAGCAGCCTTCC
*TraesCS7B03G0581800*	381059421.381062323	LRR-repeat protein	TGACGCCGTGGGAGATTGT	TTGATGCGGAGCCAGAACG
*TraesCS7B03G0812600*	545776588.545785098	transcription factor	GTGGAGCTGAGCTGAAAGG	GAAGGCATACGACCAAGAA
*TraesCS7B03G0863700*	578446982.578448448	Disease resistance protein	ACATGGCGGATACCTGAGCG	TCCGGCGTCTCGAACCACT
*TraesCS7B03G0910400*	598101444.598107287	disease resistance protein	CCGCTTATCTTAGAGGTCC	AACTCGTGCAATCCAATAC
*TraesCS7B03G0925400*	605759635.605761734	Disease resistance protein RPM1	CAAGGGTCGTTGGAGGAGT	AGAAAGCGATGGCAGTGGG
*TraesCS7B03G0959700*	622253623.622256064	Serine threonine-protein kinase	CCTCCGTAGCAGCGTAGACA	CCATTCACGTTTGCCATTTT
*TraesCS7B03G1012000*	647238432.647243000	Protein kinase domain	GGATGATGTTCCCGGTGAG	TCGGAGGCTTGGCTTTATT

## Discussion

Durum wheat is an important germplasm for wheat improvement against powdery mildew. In this study, a durum wheat accession W762 was resistant to 21 of 32 tested *Bgt* isolates, indicating that it was a valuable resistance donor for wheat improvement against powdery mildew. Genetic analysis showed that the powdery mildew resistance in W762 did not meet monogenic inheritance and complex genetic model might exist in W762. There are several reasons that may be related to this phenomenon, including alien translocation and gametocidal genes and quantitative inheritance. For example, there were evidences that the homologous group two chromosomes of *Triticeae* spp. carried genes favoring the transmission of specific gametes (Tsujimoto and Tsunewaki, 1988). Fu et al. (2013) identified a recessive *Pm* gene (*pmX*) located on chromosome arm 2AL that showed abnormal segregation. Inheritances of the two stem rust resistance genes *Sr36* and *Sr40* showed segregation distortion and recombination suppression which involved alien translocations (Tsilo et al., 2008; Wu et al., 2009). In the present study, molecular genetic analysis of F_2_ populations of the cross W762 × LDN and their derived F_2:3_ families deviated significantly from monogenic inheritance. It was supposed that the resistance to powdery mildew in W762 may be controlled by quantitative trait loci or several major *Pm* genes. Further studies on isolating single genes through multiple generations of backcrossing and self-crossing were needed in the future.

BSR-Seq is an efficient method for both rapid gene mapping and differential gene expression profiling (Wang et al., 2017; Hao et al., 2019). Using BSR-Seq, the resistance genes in W762 were postulated to be located on the intervals of 66.8–404.4 Mb and 525.7–645.1 Mb on chromosome 7B. The candidate regions have a total of 740 DEGs, and the resistance gene(s) in responding to *Bgt* invasion in W762 may be located in these candidate intervals. To further identify candidate genes/regulatory genes, we selected 18 potential targeted genes in the candidate intervals to analyze their response patterns against *Bgt* invasion. Nine of these genes did not express in both resistant parent W762 and susceptible parent LDN, and hence can be eliminated. The remaining genes were all upregulated in W762 after *Bgt* invasion, suggesting these genes may play important roles in responding *Bgt* invasion.

Plant resistance is a complex process in the course of host-pathogen interaction (Hikichi, 2016). To respond to the invasion of pathogen, a lot of genes will be activated in plants. Invasion of the pathogens could be prevented at different layers, such as cell wall, plasma membrane, and various enzymes in cytoplasm (Braeken et al., 2008; Soto et al., 2011; Siddiqui et al., 2012). In this study, following *Bgt* inoculation, a large number of DEGs, including the targeted genes in the candidate intervals, which are important for defense against *Bgt* invasion, were identified and analyzed using GO, COG, and KEGG enrichment. And three types of genes accounted for the greater proportions, including those involved in biological process, cellar component and molecular function. This result is consistent with the model of signal transduction and activation of defense mechanisms: when pathogens invade, signal transduction mechanisms are activated to transduce the stress signal; then, defense mechanisms are expected to be mobilized to fight the *Bgt* invasion; both these processes need the support of biosynthesis and metabolism.

## Conclusion

W762 is a durum wheat accession that shows high resistance to powdery mildew. We clarified the genetic pattern of powdery mildew resistance, identified DEGs at the whole-genome scale and profiled the expression of several key genes associated with resistance to powdery mildew using qRT-PCR. Our study could lay a foundation for analysis of the molecular mechanism and also provide potential targets for the improvement of durable resistance against powdery mildew.

## Data availability statement

The datasets presented in this study can be found in online repositories. The names of the repository/repositories and accession number(s) can be found in the article/supplementary material.

## Author contributions

ZQ: Writing – original draft, Writing – review & editing. RL: Writing – original draft, Writing – review & editing. XL: Writing – original draft, Writing – review & editing. YQ: Writing – original draft, Writing – review & editing. JW: Writing – original draft, Writing – review & editing. YY: Writing – original draft, Writing – review & editing. QX: Writing – original draft, Writing – review & editing. NY: Writing – original draft, Writing – review & editing. JZ: Writing – original draft, Writing – review & editing. YL: Writing – original draft, Writing – review & editing. JL: Writing – original draft, Writing – review & editing. YD: Writing – original draft, Writing – review & editing. CL: Writing – original draft, Writing – review & editing. YJ: Writing – original draft, Writing – review & editing. PM: Writing – original draft, Writing – review & editing.
